# The midwifery initiated oral health-dental service protocol: an intervention to improve oral health outcomes for pregnant women

**DOI:** 10.1186/1472-6831-15-2

**Published:** 2015-01-15

**Authors:** Maree Johnson, Ajesh George, Hannah Dahlen, Shilpi Ajwani, Sameer Bhole, Anthony Blinkhorn, Sharon Ellis, Anthony Yeo

**Affiliations:** Faculty of Health Sciences, Australian Catholic University, Ingham Institute Applied Medical Research, Sydney, Australia; Centre for Applied Nursing Research, University of Western Sydney/ South Western Sydney Local Health District, University of Sydney, Ingham Institute Applied Medical Research, Liverpool BC, Locked Bag 7103, Sydney, NSW 1871 Australia; School of Nursing & Midwifery, University of Western Sydney, Ingham Institute Applied Medical Research, Sydney, Australia; Sydney Local Health District Oral Health Services and Sydney Dental Hospital, Faculty of Dentistry, University of Sydney, Sydney, Australia; Faculty of Dentistry, University of Sydney, Sydney, Australia; Camden and Campbelltown Hospitals, South Western Sydney Local Health District, Sydney, Australia; Centre for Applied Nursing Research, University of Western Sydney, Sydney, Australia

**Keywords:** Oral health, Pregnancy, Midwives, Antenatal care, Dental

## Abstract

**Background:**

Evidence is emerging that women’s poor oral health and health practices during pregnancy are associated with poor oral health in their children and potentially an increased risk of pre-term or low-birth weight infants.

**Methods/Design:**

The Midwifery Initiated Oral Health-Dental Service (MIOH-DS) trial is a three arm multicentre randomised controlled trial which will recruit women from three metropolitan hospitals aimed at improving women’s oral health and service access and indirectly reducing perinatal morbidity. All three arms of the trial will deliver oral health promotion material, although a midwife oral assessment and referral to private/public/health fund dental services pathway (Intervention Group 1) and the midwife oral assessment and referral to local free public dental services pathway (Intervention Group 2) will be compared to the control group of oral health promotional material only. Midwives will undergo specific oral health education and competency testing to undertake this novel intervention.

**Discussion:**

This efficacy trial will promote a new partnership between midwives and dentists focused on enhancing the oral health of women and their infants. Should the intervention be found effective, this intervention, with existing on-line educational program for midwives, can be easily transferred into practice for large metropolitan health services within and beyond Australia. Further cost-benefit analysis is proposed to inform national health policy.

**Trial registration:**

Australian New Zealand Clinical Trials Registry ACTRN12612001271897.

## Background

Maintaining optimal oral health during pregnancy is now recognised as an important factor in the immediate and long-term health of women and children [1, 2]. Women are at a higher risk during pregnancy of poor oral health due to hormonal changes, dietary changes and increased nausea and vomiting [1, 3]. Poor maternal oral health has been linked to an increased risk of preterm birth and low birth weight infants [4, 5] particularly among women from lower socio-economic backgrounds [4]. Bacteria causing tooth decay can be transmitted following birth from the mother to the child though close physical contact, sharing of spoons and when mothers clean dummies with their mouths [6]. Fostering good oral health in women during pregnancy is seen as an ideal early intervention and good public health policy [7].

While some developed countries have implemented strategies to address the issue of maternal oral health during pregnancy, this has not been the case in Australia to-date [8, 9]. Only one-third of pregnant women consult a dentist when pregnant, even when oral health problems exist [9, 10]. This is partly due to the way oral health care is delivered in Australia with very limited access for low-income consumers to public dental services combined with the continuing high cost of private dental health care [11]. Even those eligible for public dental services will find themselves on lengthy waiting lists that are incompatible with the relatively short duration of pregnancy. This lack of access is also compounded by persistent, misunderstandings about the safety of dental treatment during pregnancy held by antenatal care providers, dentists and women [12].

There is mounting international evidence that midwives are the preferred health professionals to deliver the most effective maternity care for the majority of women worldwide [13–15]. As a result midwives are increasingly being supported to pass on key health messages to pregnant women, such as smoking cessation, weight control and drug and alcohol use [16]. In addition, midwives are often one of the first health professionals pregnant women encounter, and are therefore well-placed to identify and refer pregnant women early on in pregnancy when oral health problems are identified [17]. To assist midwives in delivering key messages and referrals an on-line oral health education program has been designed [18]. This education package developed and pilot-tested with midwives confirms their competence to undertake oral health care checks and provide oral health education to pregnant women [18, 19].

We now present a randomised controlled trial protocol to test the efficacy of a Midwifery Initiated Oral Health-Dental Service (MIOH-DS) in improving women’s oral health and service access and indirectly reducing infant morbidity.

### Aims and hypotheses

The aim of this study is to determine the effectiveness of the Midwifery Initiated Oral Health-Dental Service (MIOH-DS) in improving the uptake of dental services, oral health status, oral health knowledge, quality of oral health, and potentially influencing the incidence of low birth weight and preterm births in pregnant women compared to a midwife only intervention or a control group.

Pregnant women will be in one of 3 groups that will receive the following:

1. Intervention group 1 (IG1) will receive the Midwifery Initiated Oral Health (MIOH) service involving the midwifery intervention (involving oral health education, oral assessment and referrals delivered by specifically trained midwives) and referral to existing dental services (public, private or health fund clinics).
2. Intervention group 2 (IG2) will receive the MIOH-DS service which will include: a midwifery intervention (involving oral health education, oral assessment and referrals delivered by specifically trained midwives) and a dental intervention involving prompt treatment by trained dentists at specific public dental clinics.
3. The control group (CG) will not receive any midwifery or dental intervention (current practice) beyond oral health promotional material but will be provided (if required) dental referrals after reaching the end point of this trial.

#### Primary hypothesis

We hypothesise that pregnant woman attending antenatal clinics receiving MIOH-DS (IG2) or MIOH (IG1) service will have a:

- 20% increase in the access/uptake of dental services
- 30% improvement in their oral health and
- 30% increase in their knowledge about maternal oral health; compared to the control group.

## Methods/Design

### Overall study design

The MIOH-DS study is being assessed using a three arm multicentre randomised controlled trial across three recruitment sites. Participants are randomly assigned to the intervention (two groups) or control groups with a goal of n=124 in each group at the time of post intervention follow up. There are two assessment periods: baseline (12–20 weeks gestation) and post assessment (28–42 weeks gestation). The study has been funded by the National Health and Medical Research Council of Australia for three years.

### Development of the intervention

The MIOH-DS intervention has been systematically developed and piloted using a developmental framework for complex interventions [20]. Through this process the evidence base was generated [8, 18, 21], the need for an intervention was identified [22, 23] and the feasibility assessed [22, 24]. The intervention involves midwives providing oral health education, assessment and referrals to pregnant women at their first antenatal visit. To undertake this role an online oral health training package for midwives was developed [18] and tested [19] and certified by the Australian College of Midwives as a continuing professional development (CPD) activity. The training package includes an oral health screening tool that was piloted [25] and evidence-based oral health promotional material for pregnant women endorsed by the state health department [26].

### Participants and recruitment

Participants are being recruited from antenatal clinics across three large metropolitan hospitals in Greater Western Sydney, Australia. Information about the study is provided to all pregnant women attending their first antenatal visit (booking visit). At all Australian antenatal clinics the first booking visit is attended by midwives. While waiting to see the midwife, pregnant women are invited to participate by an independent recruiter (dental assistant). Women are eligible for recruitment (inclusion criteria): if they are more than 18 years of age; do not have cardiac disease that would warrant the need of antibiotics for dental treatment; have not received dental treatment in the current pregnancy; have a single pregnancy of more than 12 and less than 20 weeks of gestational age; do not have any known foetal anomalies or other risk factors (including history of preterm birth) that would place the pregnancy at risk of complications; and are able to attend regularly for dental treatment if required. Informed written consent is obtained by the dental assistant from pregnant women who meet the study inclusion criteria.

A self-administered pre-questionnaire is then provided to obtain baseline demographic data, dental/medical aspects of the women’s health (such as uptake of dental services, quality of oral health) and pre-test oral health knowledge. In addition, the Oral Health Impact Profile-14 (OHIP-14) questionnaire will also be administered. The OHIP-14 is a subjective measure of oral health that has been used extensively and found to be a precise, valid and reliable instrument (α=0.88) [27, 28]. It contains 14 questions assessed on a 5-point Likert scale and the total scores range from 0–56 with higher scores indicating poorer oral health. The OHIP-14 assists in validation and determining the sensitivity and specificity of the midwifery oral assessment tool by representing the ‘gold standard’ for identifying participants at risk of having oral health problems. Participants from non-English speaking backgrounds are provided, if required, interpreter services and translated oral health promotional materials.

### Ethics approval

This study has been approved by the Human Research Ethics Committees of Sydney Local Health District (HREC11/CRGH/289) and the University of Western Sydney (H9709).

### Randomisation

Following consent and baseline data collection participants are allocated to the groups using block randomisation. This form of randomisation is recommended for large trials [29] and has been used in a number of recent trials in this research area [21]. A permuted block randomisation scheme with a random mixture of block sizes is used to set the allocation order, stratifying participants by hospital and presence of a dental problem. Block randomisation ensures that the number of participants in each arm of the trial will be evenly balanced [29]. Randomisation of participants is accomplished centrally using an independent computerised service which is accessed via the telephone at the time of recruitment.

Group allocation (IG1, IG2, CG) is represented by coloured stickers concealed in serially numbered opaque randomisation envelopes. After recruitment, baseline data collection and randomisation via telephone, the dental assistant hands over the randomisation envelope to the pregnant women, who then gives it to the midwife. All midwives at each of the hospitals are aware of which group each coloured sticker represents. To maintain allocation concealment and minimise selection bias the dental assistants are unaware of the colour codes and block sizes used for randomisation. In addition, the study investigators are blinded to group allocation. Due to the randomisation method, pregnant women attending on the same day receive different interventions.

To minimise any distress among pregnant women and maintain equipoise [30], oral health promotion material are provided via the dental assistant at recruitment to all trial participants. Women who refuse to participate are also provided with oral health promotional material. It is also possible that women attending the antenatal clinic on the same day might interact with each other which can lead to potential contamination (estimated at <5%) in the study. We are adjusting for this contamination during analysis [31] and have also included an item in the post questionaire asking if they have discussed their particpation in the study with other women attending the antenatal clinic at the same time. Analysis is being undertaken excluding such women.

### Intervention groups

#### Intervention group 1 (IG1)

**Figure 1:**
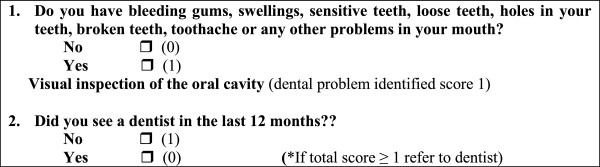
**The midwifery oral assessment tool.**

This group receives oral health promotional material at the time of recruitment. This group also receives a **midwifery intervention** (MIOH) which involves midwives providing the following at the 1^st^ antenatal visit:

- *Oral health education*: Midwives re-emphasise the importance of maternal oral health.
- *Midwifery oral assessment:* As part of the antenatal check-up, midwives (trained in oral assessment) assess the oral health status of pregnant women using the following oral assessment tool. The tool consists of two questions and a visual inspection of the oral cavity (see Figure 1).

A total score ≥ 1 for the two questions, indicates that the pregnant woman is at risk of dental problems and requires referral to a dentist. The second question is also a cue for where to refer the pregnant women (ie. private/public or health fund) and is relevant for participants in Intervention group 1 (IG1). As highlighted in our preliminary research [32] visual inspection of the oral cavity helps confirm any oral health concerns raised by the pregnant women and provides an opportunity to identify any other dental issues that may be present. The two questions have been recommended in published perinatal oral health practice guidelines [33] and utilised in previous prenatal oral health programs [34, 35]. Similar questions have also been used as a screening tool in other at-risk populations to detect poor oral health and have demonstrated adequate validity [36]. Our pilot study has shown that the tool has sufficient sensitivity to identify dental problems and facilitate referrals [25].

#### Referrals

In this group, all pregnant women assessed to be at risk of poor oral health are referred to current dental services (either private, public or health fund) for treatment. The referral letter provided to pregnant women includes a covering letter and a survey/checklist—date of first visit, total number of visits required, treatment completed (item numbers), and dentists contact details—to be completed by the dentists and returned to the study investigators via fax. Information on the survey/checklist is also sought from private dentists whose contact details are provided by pregnant women in the post questionnaire. The checklist helps to assess the uptake of dental services, the common treatment requirements and associated dental costs, all of which are vital information to inform future economic evaluations and subsequent state and national policy in this area.

##### Intervention group 2 (IG2)

This group receives oral health promotional material at the time of recruitment. This group also receives a **midwifery and dental intervention** (MIOH-DS). The **midwifery intervention** will be similar to Intervention group 1. However, in this group all pregnant women, regardless of whether they are assessed to be at risk of having dental problems or not by the midwife, are referred (referral letter with checklist) to the dentists employed for this study for an initial dental oral assessment (part of the dental intervention). This allows for further testing of the midwifery oral assessment tool and to gather baseline oral health data. Pregnant women who consent are provided vouchers that entitle them to priority access to free oral assessment and treatment by the study dentists at dental clinics in the recruitment hospitals. This service is an extra service created specifically for this study and is co-ordinated by the study team. The voucher is valid for 4 weeks from booking visit date to encourage pregnant women to see the dentist promptly. Pregnant women can make the dental appointment by calling the telephone number provided with the referral letter or contacting the dental assistant in the antenatal waiting room.

##### Dental intervention

At the first appointment all the women undergo an initial dental oral assessment that includes medical history, followed by oral mucosal tissue examination, periodontal examination, dental caries examination and denture evaluation. A provisional diagnosis and treatment plan is made for those women having oral health problems. Once the consent for treatment is obtained, subsequent appointments are made in the second trimester to complete all the urgent treatment for women having dental problems. The second trimester (13–27 weeks) is considered a safe period to carry out necessary dental treatment^10^. The completion of treatment will take 1 to 3 additional visits depending on the treatment plan. Women having complex treatment needs such as root canal treatment are referred to the specialist for follow up care and are excluded from the study. After each examination participants are provided oral health education including oral hygiene instructions, brushing and flossing instructions and dietary counselling.

All women in the control group, intervention group 1 and group 2 (whether or not at risk of dental problems) undergo a final dental oral assessment by the study dentists at 28–38 week gestation period, the end point of this study. This examination is similar to the initial dental oral assessment and includes a detailed clinical examination. All women receive reminder cards from midwives followed up by telephone calls from the study administrative assistant to attend their final dental oral assessment. Pregnant women in the control group identified with a dental problem are referred to dental services after pregnancy. A post questionnaire having similar items to the pre questionnaire (demographic data, dental/medical aspects of the women’s health such as uptake of dental services, quality of oral health and post-test oral health knowledge), is administered to all women between 28–32 weeks. Contact details of private dentists who were consulted during pregnancy are also sought in the post questionnaire. The following methods are used to administer the post questionnaires: via the dental assistant/receptionist in the antenatal waiting room, via the midwife during the antenatal visit, over the phone when the final dental appointments are being made and via the study dentists at the final dental appointment.

##### Education for midwives and dentists

Prior to the commencement of the trial all midwives from participating hospitals undertake the specific oral health education program endorsed by the Australian College of Midwives [18]. Midwives have to complete the program and competency assessment to ensure that they have adequate knowledge of oral health and are competent to undertake an oral visual inspection. Prior to the trial the two study dentists employed for the study participate in a two hour education workshop where they are trained by an experienced dental clinician to follow a standardised dental protocol during pregnancy. Training is also provided on the use of the oral health status measures. Inter-rater reliability testing of the dentists’ oral health assessments will be examined using 10 mock oral assessments.

##### Control group (CG)

**Figure 2:**
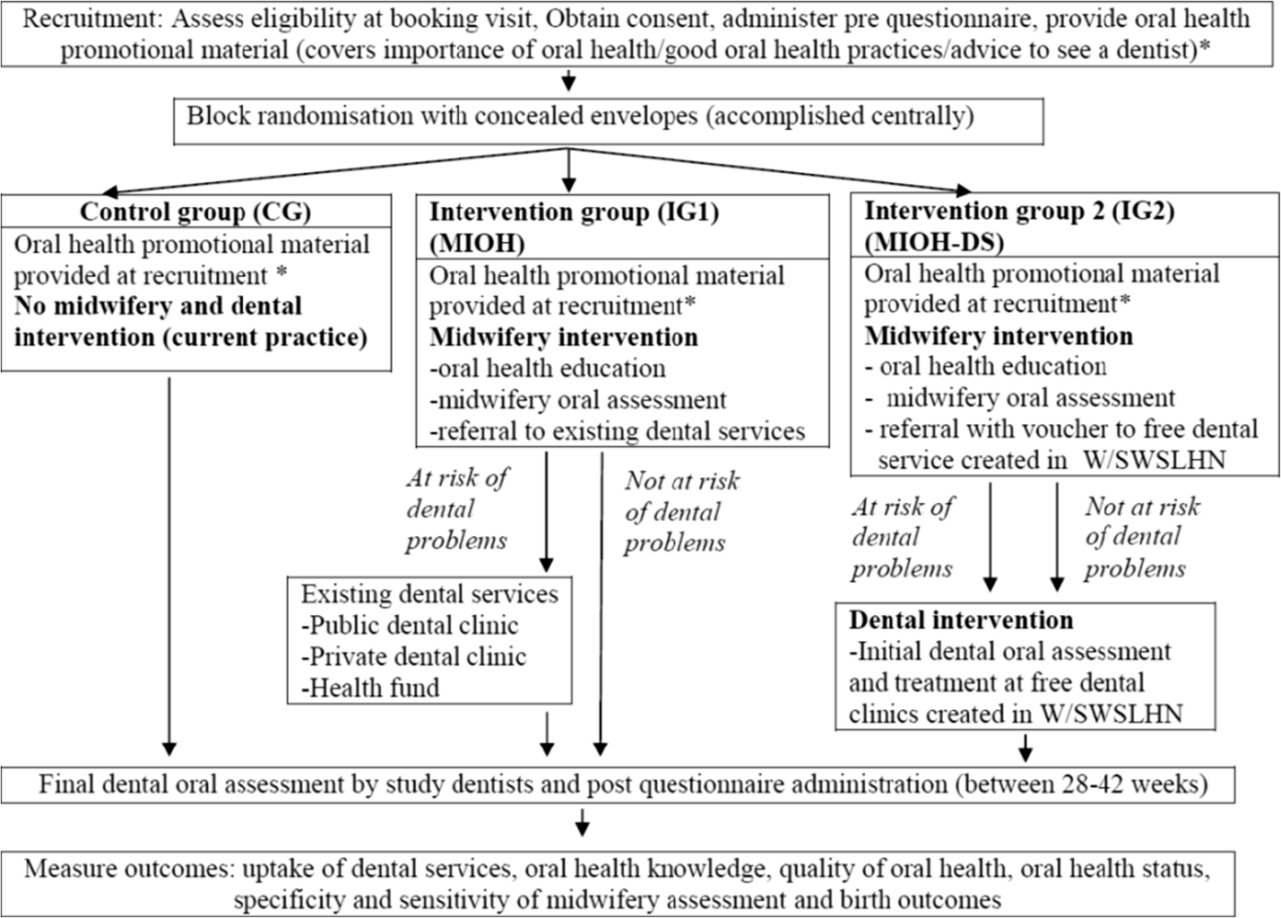
**Detailed design of MIOH-DS trial.**

This group receive oral health promotional material at recruitment. This material focuses on the importance of oral health, good oral health practices (dietary counselling, fluoride use, quitting smoking) and advice on seeing a dentist during pregnancy. They do not receive any intervention until the completion of the trial. At the point of completion, these women are referred to dental services, if required. Figure 2 outlines the design of the MIOH-DS trial which follows the principle of equipoise.

### Outcome measures

The following outcome measures—estimates of uptake of dental services, oral health knowledge, quality of oral health, oral health status and birth outcomes are proposed for this trial:

- *An estimate of the uptake of dental services* will be obtained from the following sources: the returned checklist; the database of the study dental clinics; data from the post questionnaire, following up all referrals by midwives and at the final dental oral assessment period; contacting private/health fund dentists used by the women (contact details obtained from the post questionnaire and returned checklist).
- *Oral Health Knowledge* will be assessed using a pre-test and post-test questionnaire (tested in the pilot study) [19] and administered to pregnant women at the recruitment and final dental oral assessment period respectively.
- *Quality of oral health* will be assessed using the following validated item [37] to be included in the pre and post questionnaire: ‘On a scale of 1–5 where 1= poor and 5=excellent, How would you describe the health of your teeth and mouth?
- *Oral health status* will be assessed using several measures—gingival inflammation and bleeding, clinical attachment loss, level of plaque and dental caries*.* These measures are to be used at both the dental oral assessments:
  –Gingival inflammation and bleeding is assessed using The Sulcus Bleeding Index [38] and is used to measure the degree of inflammation and bleeding in the gums;
  –Clinical Attachment Loss (CAL), measures the degree of CAL (periodontal pocket depth, gingival recession and calculus) and is assessed using a calibrated periodontal probe [39];
  –Level of plaque is measured using The Approximal Plaque Index [38].
  –Dental caries will be determined by the Decayed, Missing, Filled Teeth (DMFT) Index [40]. The DMFT index is used to monitor the changes in the various components. If the intervention is successful decayed teeth will have been filled or removed.

- *Birth outcomes*: Gestational age at birth and birth weight is obtained from the ObstetriX data system (ODS) [41] which is a reliable data source (Kappa =0.75) using data items/definitions from the NSW Perinatal Data Collection (population based surveillance system covering all births in NSW public/private hospitals). Data from ODS is accessed using the women’s medical record numbers and date of birth (collected at recruitment) through local data custodians.
- *Specificity and sensitivity of midwifery assessment:* To further confirm the practicality of this tool, the midwifery oral assessment tool (2 items) will be compared with two gold standard measures- the OHIP-14 questionnaire (completed at the time of recruitment: pre-questionnaire) in IG1 and IG2 and the initial dental oral assessment conducted by the two study dentists in IG2. This will allow for women with and without oral health problems (the proportion of true and false positives and the proportion of true and positive negatives) to be identified. The OHIP-14 questionnaire has been used as the gold standard in previous studies to validate oral assessment tools [36, 42]. The two study dentists are trained to follow a standardised dental protocol during pregnancy. Adherence to the protocol is being evaluated by assessing the returned checklist.

### Sample size

Sample size estimation is calculated on the outcome measure uptake of dental services using the assumption that 30% of pregnant women in the control group will access dental services during pregnancy (based on our survey [23] and existing Australian studies [10, 43, 44]. We expect the MIOH (IG1) and MIOH-DS service (IG2) will improve the uptake of dental services. The MIOH intervention is considered successful if at least 50% of the pregnant women in IG1 and IG2 will access dental services during pregnancy, a difference of 20% between the groups. To detect a difference of at least 20% between the groups with a two-sided test of proportions, alpha of 0.017 (three comparisons 0.05/3), and 80% power we determine using a statistical software that we require at least 124 patients in each group. Taking into account 10% attrition (refusal after randomisation-based on the results of our pilot survey) and 30 % loss at the endpoint (based on trials in this area - range 2-30%) [21] we will need to recruit 207 participants in each group.

Sample size was also calculated on the outcome measure oral health status using the assumption that 50% of pregnant women in the control group will have gingival bleeding (based on trials in this area) [21]. A 30% improvement in oral health status is expected in IG1 and IG2 (30% reduction in gingival bleeding). To detect a difference of at least 30% between the groups with a two-sided test of proportions, alpha of 0.017, and 80% power we determine using statistical software [45] that we require at least 52 patients in each group. Taking into account 10% attrition and 30 % loss at the endpoint we will need to recruit 87 participants in each group. Based on these estimations we will require a total of 621 participants for the trial (207 in each group). Interim analyses will be used to re-check the sample size after the first 100 women are recruited.

### Data analysis

Per-protocol and intention-to-treat analysis will be conducted by staff blinded to the group allocation. Missing data will be coded in the database structure and handled using an appropriate method. Demographic data will be analysed using descriptive statistics. All variables will be examined for normality and appropriate data transformations will be applied. Potential confounders (income, pre-existing oral health conditions, and others) and the impact of the MIOH/MIOH-DS intervention will be examined using univariate and multivariate methods. Regression analysis that take into account the variables of interests to the outcome will be performed. Some of the variables will be considered as exerting fixed effects (e.g. the number of patient groups) while others as random effects e.g. oral health knowledge. The regression models will also include a hospital variable (1–3). Analysis will be conducted for each hospital clinic and if no differences are found, data will be pooled. Pearson’s Chi-square test will be used to analyse the proportion of pregnant women accessing dental services. Oral health status and knowledge outcomes will be analysed using Analysis of Variance and post-hoc procedures. The effectiveness of the oral health education program will be assessed using dependent t-tests.

The sensitivity and specificity of the oral assessment tool using the OHIP-14 as the gold standard will be calculated using odds ratios. For this analysis the total scores using the midwifery oral assessment tool for participants in IG1 and IG2 will be dichotomised as 0=0 (no risk) and 1 to 2=1 (at risk). The total scores for participants using the OHIP-14 in IG1 & 2 will also be dichotomised using the median split as described by Locker et al. [42]. Subjects with an OHIP-14 score of 0–7 will be scored 0 (no risk) and 8–56 a score of 1 (at risk). Sensitivity and specificity will also be calculated using the initial dental oral assessment in IG2 as the gold standard. Analysis of proportions using chi square procedures (two tailed) and confidence intervals will be undertaken to determine sensitivity (true positives), specificity (true negatives), false positives, false negatives, negative and positive predictive values [46].

## Discussion

Oral health during pregnancy continues to remain a poorly assessed and treated aspect of the health of all Australians [8]. The Australian Government continues to poorly fund oral health iniatives in this area even though evidence of escalating incidence of poor oral health in children (40% have dental caries by age 5–6 years) continues [6]. This study represents a ground-breaking oral health initiative that has the potential to deliver a cost-effective oral health intervention during pregnancy delivered by a unique partnership of health professionals.

We have developed a novel intervention to increase access to public/private dental services for pregnant women, with the potential to improve women’s oral health status and potentially indirectly reduce infant morbidity. The unique combination of midwives and dentists, appropriately educated in oral assessment techniques and the specialist needs of pregnant women, acting together in this intervention, is an international first and represents a profound shift in our understanding of which health professionals are responsible for oral health. The benefits to women and infants, particularly those women experiencing financial hardship as a barrier to accessing dental services, have been established [8, 43] and continues to unfold [47–49]. The targeting of midwives, as the first point of health professional contact for many Australian pregnant women, to increase awareness of the consequences of poor oral health during pregnancy, and refer women to dental services, may provide added encouragement to women to attend dental services not seen in other international oral health strategies targeting pregnant women [50]. Dispelling existing misinterpretations of the safety of undergoing dental treatments during pregnancy [51, 52] may be an unexpected outcome as challenges to these myths occur through oral health promotional material and attendance of women at private dental clinics during this trial. Although the primary focus of this trial is on the oral health of pregnant women, infant morbidity will be determined throughout this study and compared to other larger Australian trials specifically addressing infant morbidity following dental interventions [49].

This multicentre randomised controlled trial, with participant and researcher concealment, will provide initial evidence of the efficacy of this intervention for large health services throughout Australia or elsewhere. The intervention, if found effective, will readily translate into clinical guidelines and policy for further national consensus. Data are also being captured to undertake a cost-benefit analysis to support subsequent lobbying of state or federal Australian governments for this initiative. This initiative—midwife assessessment and referral to dental services [vouchers and/or localised dental services]-may represent a cost-effective oral health policy that appropriately targets and long-term improves the oral health of Australian children.

## Abbreviations

MIOH: Midwifery initiated oral health
MIOH-DS: Midwifery initiated oral health- dental service
IG2: Intervention group 2
IG1: Intervention group 1
C: Control group
OHIP-14: Oral health impact profile-14
CPD: Continuing professional development.

## References

[1] Silk H, Douglas AB, Douglas JM, Silk L (2008). Oral health during pregnancy. Am Fam Physician.

[2] Steinberg BJ, Hilton IV, Iida H, Samelson R (2013). Oral health and dental care during pregnancy. Dent Clin N Am.

[3] Klepacz-Szewczyk J, Pawlicka H (2014). Most frequent oral pathological states problems occurring in pregnant patient. Dent Med Probl.

[4] Xiong X, Buekens P, Fraser WD, Beck J, Offenbacher S (2006). Periodontal disease and adverse pregnancy outcomes: a systematic review. BJOG.

[5] Ide M, Papapanou PN (2013). Epidemiology of association between maternal periodontal disease and adverse pregnancy outcomes–systematic review. J Clin Periodontol.

[6] Health N (2009). Early childhood oral health guidelines for child health professionals 2nd edition.

[7] Yost J, Li Y (2008). Promoting oral health from birth through childhood: prevention of early childhood caries. MCN Am J Matern Child Nurs.

[8] George A, Johnson M, Blinkhorn A, Ellis S, Bhole S, Ajwani S (2010). Promoting oral health during pregnancy: current evidence and implications for Australian midwives. J Clin Nurs.

[9] George A, Ajwani S, Bhole S, Johnson M, Blinkhorn A, Ellis S (2010). Promoting perinatal oral health in South-Western Sydney: a collaborative approach. 2010;89 (special issue C). J Dent Res.

[10] Thomas N, Middleton P, Crowther C (2008). Oral and dental health care practices in pregnant women in Australia: a postnatal survey. BMC Pregnancy Childbirth.

[11] New South Wales Health (2009). Oral health—eligibility of persons for public oral health care in NSW (PD2009_074).

[12] Wasylko LMD, Dykxhoorn SM, Rieder MJ, Weinberg S (1998). A review of common dental treatments during pregnancy: implications for patients and dental personnel. J Can Dent Assoc.

[13] Renfrew MJ, Homer CSE, Downe S, McFadden A, Muir N, Prentice T (2014). The Lancet’s series on midwifery executive summary. Lancet.

[14] ten Hoope-Bender P, de Bernis L, Campbell J, Downe S, Fauveau V, Fogstad H (2014). Improvement of maternal and newborn health through midwifery. Lancet.

[15] Van Lerberghe W, Matthews Z, Achadi E, Ancona C, Campbell J, Channon A (2014). Country experience with strengthening of health systems and deployment of midwives in countries with high maternal mortality. Lancet.

[16] Edvardsson K, Ivarsson A, Garvare R, Eurenius E, Lindkvist M, Mogren I, *et al*.: **Improving child health promotion practices in multiple sectors – outcomes of the Swedish Salut Programme.** *BMC Public Health* 2012.,**12**(920)**:** doi:10.1186/1471–2458–1112–1920 10.1186/1471-2458-12-920 PMC3564907 23107349

[17] George A, Johnson M, Blinkhorn A, Ajwani S, Ellis S, Bhole S (2014). Views of pregnant women in South Western Sydney towards dental care and an oral-health program initiated by midwives. Health Promot J Austr.

[18] George A, Duff M, Ajwani S, Johnson M, Dahlen H, Blinkhorn A (2012). The development of an education program for midwives in Australia to improve perinatal oral health. J Perinat Educ.

[19] George A, Duff M, Johnson M, Dahlen H, Blinkhorn A, Ellis S (2014). Piloting of an oral health education programme and knowledge test for midwives. Contemp Nurse.

[20] Craig P, Dieppe P, Macintyre S, Michie S, Nazareth I, Petticrew M (2008). Developing and evaluating complex interventions: the new Medical Research Council guidance. Br Med J.

[21] George A, Shamim S, Johnson M, Ajwani S, Bhole S, Blinkhorn A (2011). Periodontal treatment during pregnancy and birth outcomes: a meta-analysis of randomised trials. Int J Evid Based Healthc.

[22] George A, Johnson M, Blinkhorn A, Ajwani S, Ellis S, Bhole S (2013). Views of pregnant women in South Western Sydney towards dental care and an oral-health program initiated by midwives. Health Promot J Austr.

[23] George A, Johnson M, Blinkhorn A, Ajwani S, Bhole S, Yeo AE (2013). The oral health status, practices and knowledge of pregnant women in south-western Sydney. Aust Dent J.

[24] George AJ, Ellis M, Dahlen S, Blinkhorn H, Bhole A, Ajwani S (2010). Promoting dental health in pregnant women: a new role for midwives in Australia. Aust Nurs J.

[25] George A (2014). Developing and testing of a midwifery oral health screening tool for pregnant women. Health Care Women Int.

[26] Health N (2010). Keep smiling while you are pregnant.

[27] Slade GD, Ghezzi EM, Heiss G, Beck JD, Riche E, Offenbacher S (2003). Relationship between periodontal disease and C-reactive protein among adults in the atherosclerosis risk in communities study. Arch Intern Med.

[28] Allen PF, McMillan AS, Locker D (2001). An assessment of sensitivity to change of the oral health impact profile in a clinical trial. Community Dent Oral Epidemiol.

[29] Beller EM, Gebski V, Keech AC (2002). Randomisation in clinical trials. Med J Aust.

[30] Ashcroft RE (2004). Ethics of randomised controlled trials-not yet time to give up on equipoise. Arthritis Res Ther.

[31] Torgerson DJ (2001). Contamination in trials: is cluster randomisation the answer?. BMJ.

[32] George A, Johnson M, Duff M, Ajwani S, Ellis S (2010). Maintaining oral health durign pregnancy: perceptions of midwives in Southwest Sydney. Collegian.

[33] Health NYSDo (2006). Oral health care during pregnancy & early childhood: practice guidelines.

[34] Stevens JIH, Ingersoll G (2007). Implementing an oral health program in a group prenatal. Practice. J Obstet Gynecol Neonatal Nurs.

[35] Carl LDRGMR (2000). Exploring dental hygiene and perinatal outcomes. AWHONN Lifelines.

[36] Jeganathan S, Purnomo J, Houtzager L, Batterham M, Begley K (2010). Development and validation of a three‒item questionnaire for dietitians to screen for poor oral health in people living with human immunodeficiency virus and facilitate dental referral. Nutr Dietetics.

[37] Locker D, Jokovic A, Clarke M (2004). Assessing the responsiveness of measures of oral health‒related quality of life. Community Dent Oral Epidemiol.

[38] Klages U, Weber AG, Wehrbein H (2005). Approximal plaque and gingival sulcus bleeding in routine dental care patients: relations to life stress, somatization and depression. J Clin Periodontol.

[39] Newman MG TH, Carranza FA (2002). Carranza’s clinical periodontology.

[40] Aggeryd T (1983). Goals for oral health in the year 2000: cooperation between WHO, FDI and the national dental associations. Int Dent J.

[41] Taylor L, Pym M, Bajuk B, Sutton L, Travis S, Banks C (2000). New South Wales mothers and babies 1998. N S W Public Health Bull.

[42] Locker D, Matear D, Stephens M, Lawrence H, Payne B (2001). Comparison of the GOHAI and OHIP‒14 as measures of the oral health‒related quality of life of the elderly. Community Dent Oral Epidemiol.

[43] Keirse MJNC, Plutzer K (2010). Women’s attitudes to and perceptions of oral health and dental care during pregnancy. (Author abstract) (Report) (Survey). J Perinat Med.

[44] Ressler-Maerlender J, Krishna R, Robison V (2005). Report from the CDC. Oral health during pregnancy: current research. Journal of Women’s Health (15409996).

[45] (SPSS) SPSS (2004). SamplePower Version 2.0.

[46] Altman DG, Bland JM (1994). Diagnostic tests 1: sensitivity and specificity. BMJ.

[47] Shub A, Wong C, Jennings B, Swain J, Newnham J (2009). Maternal periodontal disease and perinatal mortality. Aust N Z J Obstet Gynaecol.

[48] Meyer K, Geurtsen W, Günay H (2010). An early oral health care program starting during pregnancy. Clin Oral Investig.

[49] Newnham J, Newnham IA, Ball CM, Wright M, Pennell CE, Swain J (2009). Treatment of periodontal disease during pregnancy a randomized controlled trial. Obstet Gynecol.

[50] *What are my rights during pregnancy?*. http://www.nhs.uk/chq/Pages/953.aspx?CategoryID=54&SubCategoryID=138#close

[51] District I, Obstetricians ACo, Gynecologists (2010). Oral health during pregnancy and early childhood: evidence-based guidelines for health professionals. J Calif Dent Assoc.

[52] Detman LA, Cottrell BH, Denis-Luque MF (2010). Exploring dental care misconceptions and barriers in pregnancy. Birth.

[CR53] The pre-publication history for this paper can be accessed here:http://www.biomedcentral.com/1472-6831/15/2/prepub

